# Iron overload may be critical for liver dysfunction in anorexia nervosa, and the role of haematocrit-adjusted albumin in assessing nutritional status: a case report

**DOI:** 10.1186/s12887-023-04367-6

**Published:** 2023-10-31

**Authors:** Tomohiko Yoshida, Toshiki Namiki, Masaya Yamaga, Shunichiro Onishi, Minoru Takemoto

**Affiliations:** Department of Diabetes, Metabolism, and Endocrinology, International University of Health and Welfare, Narita Hospital, 852 Hatakeda, Narita-shi, Chiba 286-8520 Japan

**Keywords:** Anorexia Nervosa, Liver dysfunction, iron overload, Hematocrit-adjusted albumin, Nutritional marker

## Abstract

**Background:**

Anorexia nervosa (AN) is frequently associated with liver dysfunction, but the precise mechanism remains undefined. Since the nutritional marker albumin has a low correlation with changes in body weight in AN, and patients with AN often have dehydration as a complication, we also examined whether haematocrit (HCT)-adjusted serum albumin could be a better nutritional marker in AN.

**Case presentation:**

We describe a 15-year-old girl with severe weight loss and liver damage whose liver enzymes normalized after 1.5 months of hospitalization and weight gain. We found a significant correlation between body weight (BW) and HCT-adjusted serum albumin (Spearman’s rank correlation coefficient (r_s_) = 0.66, *P* = 5.28 × 10^−3^) and between BW and alanine aminotransferase (ALT) (r_s_ = -0.825, *P* = 8.45 × 10^−5^). After division by HCT, correlations between serum albumin and ALT (r_s_ = -0.835, *P* = 5.24 × 10^−5^) and between the iron-storage protein ferritin and the liver enzyme gamma-glutamyl transferase (r_s_ = 1.0, *P* = 0.017) were also statistically significant.

**Conclusion:**

These results suggest that improvement of the nutritional status in AN could relieve liver dysfunction and facilitate iron transport. Since a decrease in the iron-transport protein transferrin presumably increases labile non-transferrin-bound iron, resulting in excess reactive oxygen species production, a defect in iron transport due to malnutrition could be one of the causes of liver injury in AN. In addition, HCT-adjusted albumin could be a better marker than its raw data to assess changes in nutritional status in AN.

## Background

Anorexia nervosa (AN) is often associated with liver dysfunction. Elevated aspartate aminotransferase (AST) and alanine aminotransferase (ALT) levels are found in 30% of the restricting type and 7.3% of the binge eating type [1]. Elevations can occur during malnutrition and also reportedly occur in one-third of patients with AN during refeeding [2]. Some reports attribute the elevations to hepatocyte autophagy and fat deposition, but the detailed mechanism remains unclear [3, 4]. We present a patient with AN with elevated liver enzymes, ferritin, and transferrin saturation (TSAT). Her ALT and gamma-glutamyl transferase (GGT) decreased with nutritional improvement in association with TSAT and ferritin reduction. Studies suggest that repeated blood transfusions may cause an iron overload (IOL), increasing labile non-transferrin-bound iron (NTBI), followed by reactive oxygen species (ROS) production, resulting in heart and liver damage [5]. We hypothesized that malnutrition-induced low transferrin levels in AN might cause IOL and hepatic injury. This study investigated the relationship between liver enzymes and nutritional indices or iron metabolism markers in a patient with restricting-type AN.

Blood albumin (Alb) and pre-albumin are known as nutritional markers; however, a recent systematic review has suggested that they may not reflect nutritional status in malnourished patients without inflammatory diseases [6]. However, since restricting-type AN is often complicated by dehydration, we hypothesized that we could use these markers if we could eliminate the effects of dehydration. Since haematocrit (HCT) has been generally used to estimate plasma volume [7], we also examined whether the value obtained by dividing Alb by HCT (Alb/HCT) correlates with body weight (BW).

## Case presentation

A 15-year-old girl was hospitalized with severe weight loss and difficulty standing due to restricting-type AN. She weighed 51.2 kg (body mass index [BMI] 20.5 kg/m^2^) a year before admission but was jealous of her thin friend and tried to lose weight through intensive exercise and calorie restriction. Owing to her continuous weight loss, she was referred to the psychiatric department of our hospital, weighing 28.7 kg (11.42 kg/m^2^) on admission; she was further referred to our department for medical stabilization. Her medical history was unremarkable, and she had been taking vitamin B complex, including benfotiamine 75 mg/day, pyridoxine 75 mg/day, and cyanocobalamin 0.75 mg/day to prevent vitamin B1, B6, and B12 deficiencies, which had been prescribed by her previous doctor, for three months before admission.

On day 3, her test results showed hypoglycaemia and elevated liver enzymes, TSAT, ferritin, and blood urea nitrogen (BUN)/creatinine (Cre) ratio. However, her serum iron and electrolytes were within normal limits (Table 1 A). Her endocrine tests showed typical findings of AN, including stress-induced hypercortisolaemia, low triiodothyronine and thyroxine levels with normal thyroid-stimulating hormone indicating euthyroid sick syndrome, high growth hormone levels accompanying low insulin-like growth factor-1, and hypogonadotropic hypogonadism (Table 1B).

**Table 1:** The patient’s test results on day 3 of admission. The patient’s complete blood count and blood chemistry (A) and endocrinological test results (B) on day three are shown

	Patient’s value	Reference range
**A**		
WBC count (/µL)	4.34 × 10^3^	3.30–8.60 × 10^3^
RBC count (/µL)	4.75 × 10^6^	3.86–4.92 × 10^6^
Hgb (g/dL)	14.0	11.6–14.8
HCT (%)	40.9	35.1–44.4
PLT count (/µL)	111.0 × 10^3^	158–348 × 10^3^
Albumin (g/dL)	4.6	4.1–5.1
BUN (mg/dL)	46.7	8–20
Creatinine (mg/dL)	1.03	0.46–0.79
T-Bil (mg/dL)	1.7	0.4–1.5
AST (U/L)	176	13–30
ALT (U/L)	170	7–23
GGT (U/L)	56	9–32
ChE (U/L)	164	201–421
CK (U/L)	253	41–153
Sodium (mEq/L)	135	138–145
Potassium (mEq/L)	4.4	3.6–4.8
Chloride (mEq/L)	103	101–108
Calcium (mg/dL)	9.1	8.8–10.1
Phosphorus (mg/dL)	2.9	2.8–4.6
Magnesium (mg/dL)	2.4	1.8–2.6
T-Cho (mg/dL)	334	150–219
Iron (µg/dL)	163	40–188
TIBC (µg/dL)	208	246–410
TSAT (%)	78.4	20–50
Ferritin (ng/mL)	726	5–152
Glucose	68	73–109
**B**		
ACTH (pg/mL)	26.7	7.2–63.3
Cortisol (µg/dL)	29.1	6.24–18.0
DHEA-S^†^ (µg/dL)	330	51–321 (18–20 years, female)
TSH (µIU/mL)	4.06	0.50–5.00
fT3 (pg/mL)	<0.4	2.3–4.0
fT4 (ng/dL)	0.8	0.9–1.7
GH (ng/mL)	96	96
IGF-1 (ng/mL)	<7	192–614 (15 years, female)
LH (mIU/mL)	<0.3	1.76–10.24 (follicular, female)
FSH (mIU/mL)	<0.3	3.01–14.72 (follicular, female)
Estradiol (pg/mL)	11.5	28.8–196.8 (follicular, female)

*Abbreviations*: *ACTH* Adrenocorticotropic hormone, *ALT*, Alanine aminotransferase, *AST* Aspartate aminotransferase, *BUN* Blood urea nitrogen, *ChE* Cholinesterase, *CK* Creatine kinase, *DHEA-S* Dehydroepiandrosterone sulphate, *FSH* Follicle-stimulating hormone, *fT3* Free triiodothyronine, *fT4* Free thyroxine, *GGT* Gamma-glutamyl transpeptidase, *GH* Growth hormone, *HCT* Hematocrit, *Hgb* Haemoglobin, *IGF-1* Insulin-like growth factor 1, *LH* Luteinizing hormone, *PLT* Platelet, *RBC* Red blood cell, *T-Bil* Total bilirubin, *T-Cho* Total cholesterol, *TIBC* Total iron binding capacity, *TSAT* Transferrin saturation, *TSH* Thyroid-stimulating hormone, *WBC* White blood cell

^†^There are no DHEA-S reference ranges for 15-year-old females available, so the ranges for 18 to 20-year-old females are shown

**Fig. 1:**
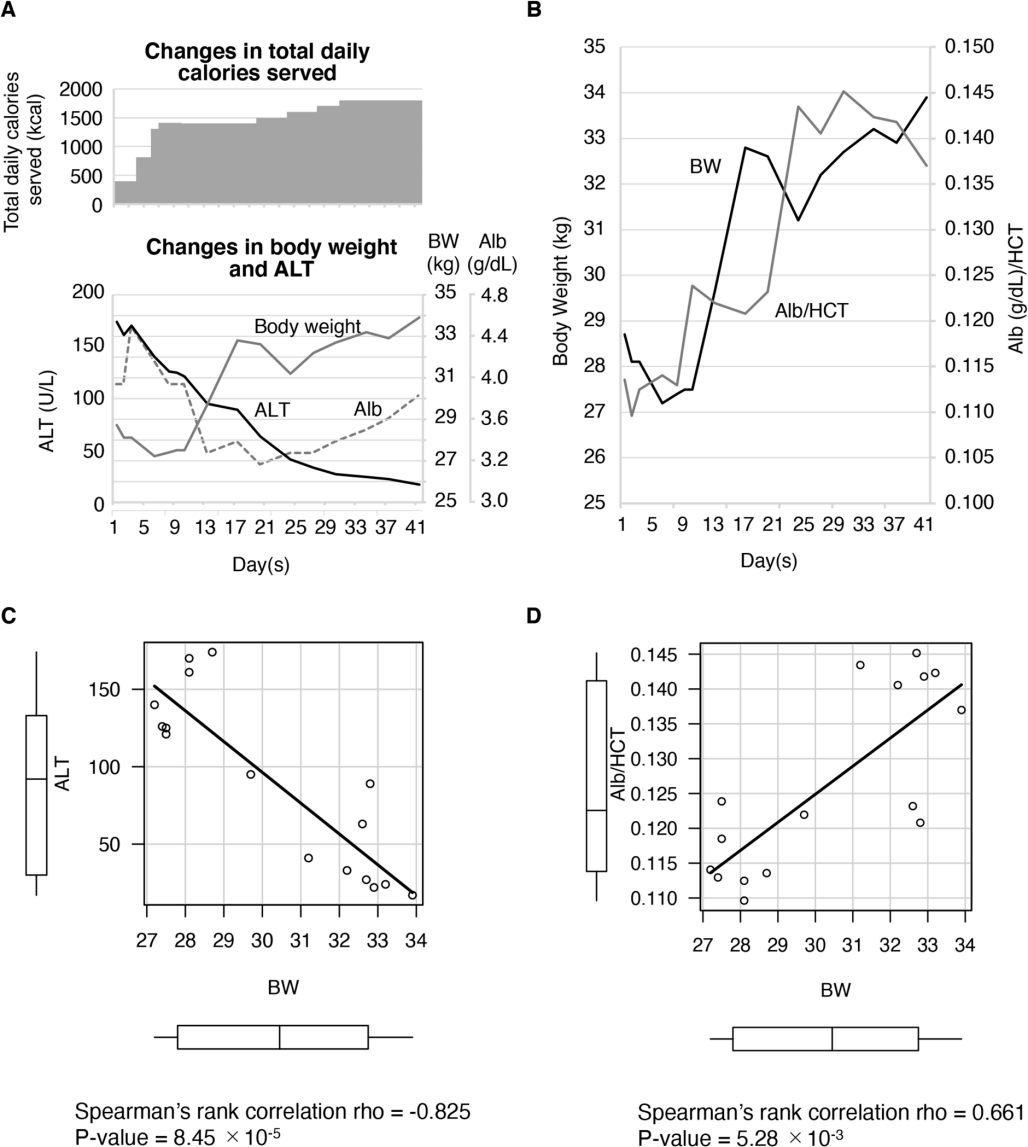
Time course and scatterplots of alanine aminotransferase (ALT) and nutritional parameters during hospitalization. **A**, **B**. Time course of nutritional indicators and the patient’s test results. **A** Changes in total daily calories (kcal) served to the patient (top), body weight (BW, kg) (bottom, grey solid line), albumin (Alb, g/dL) (bottom, grey dotted line), and ALT(U/L) (bottom, black solid line). **B** The time course of BW (solid black line) and hematocrit-adjusted Alb (Alb/HCT, solid grey line). We divided Alb by HCT to adjust for concomitant dehydration **C**, **D**. Scatterplots and nonparametric test results of BW versus ALT or Alb/HCT. Figures C and D indicate the relationship between BW and ALT and between BW and Alb/HCT, respectively. The box plots on the X and Y axes show the data distribution. The box indicates the interquartile range (IQR), and the solid horizontal line represents the median. Whiskers are expanded to the most extreme data point of no more than 1.5 × IQR from the edge of the box. Open circles reveal outliers. Nonparametric test results are shown below each scatterplot. For the statistical analysis, we used free statistical software R version 4.2.1 (The R Foundation for Statistical Computing Platform, https://www.r-project.org)

Initially, she would spit out food, but a few days later, she began to eat the whole amount. As she was able to consume the entire diet, we increased the total calories of the diet (Fig. 1A, top). One week later, her weight began to increase, and on day 44, she weighed 35.2 kg (BMI 14.0 kg/m^2^), with liver enzyme improvements (Fig. 1A, bottom). A non-parametric test determining the correlation between weight and ALT showed a significant correlation (*r*_*s*_ = -0.816, *P* = 1.16 × 10^−4^) (Fig. 1C). By contrast, her serum Alb peaked when her BW was around its lowest and reached the minimum right after her rapid weight gain two weeks after hospitalization (Fig. 1A, bottom). Therefore, as reported elsewhere, Alb itself appeared to be unrelated to the change in BW [6].

As shown in Fig. 1A, blood Alb appeared to have a low correlation with BW. Regardless, since patients with AN tend to be dehydrated, and the patient’s BUN/Cre ratio was high on day 3 (Table 1A), we realized that dehydration might elevate her blood test parameters; this could cause us to misinterpret her nutritional status. When we divided Alb values by HCT (Alb/HCT) to correct for the effects of dehydration, we found a trend toward an increase in Alb/HCT along with weight gain (Fig. 1B). A non-parametric test determining the correlation between Alb/HCT and BW indicated a significant correlation with a Spearman’s rank correlation coefficient (r_s_) of 0.661 (*P* = 5.28 × 10^−3^) (Fig. 1D). Thus, we decided to use Alb/HCT as a surrogate marker for nutritional status in this study.

**Fig. 2:**
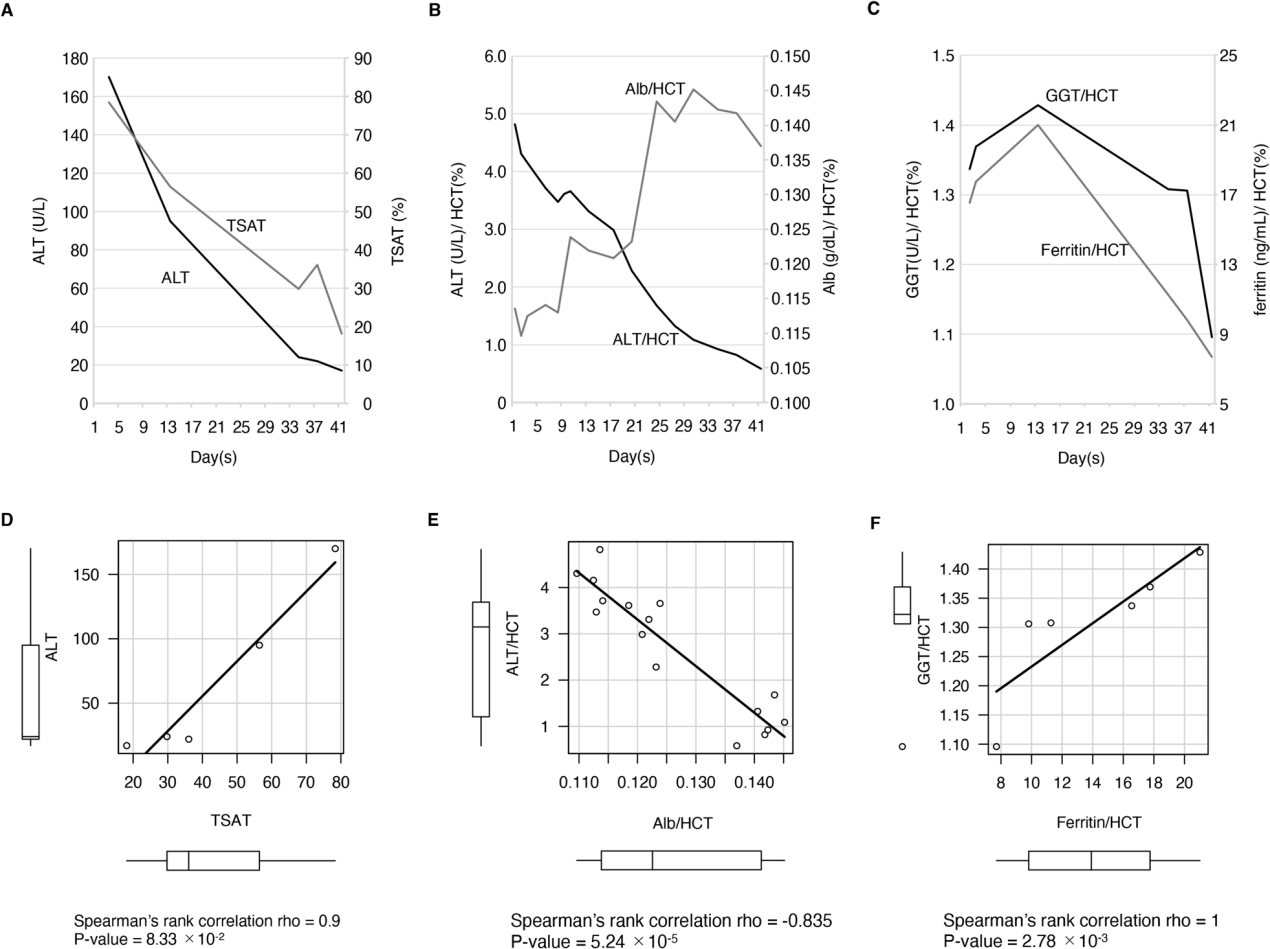
Time course and scatterplots of liver enzymes, Alb/HCT, and markers of iron metabolism during hospitalization. **A**,** C** Time course of the patient's test results. **A** Changes in transferrin saturation (TSAT, %) and alanine aminotransferase (ALT, IU/L). **B** ALT (U/L) and albumin (Alb, g/dL) change during the course. **C** Changes in GGT (U/L) and ferritin (ng/mL). For Figures B and C, each parameter was divided by hematocrit (HCT, %) to adjust for concomitant dehydration. **D**–**F** Scatterplots and nonparametric test results. Figures **D**, **E**, and **F** indicate the relationship between TSAT and ALT, Alb/HCT and ALT/HCT, and GGT/HCT and ferritin/HCT, respectively. The box indicates the interquartile range (IQR), and the solid horizontal line represents the median. Whiskers are expanded to the most extreme data point of no more than 1.5 × IQR from the edge of the box. Open circles reveal outliers. Nonparametric test results are shown below each scatterplot. For the statistical analysis, we used free statistical software R version 4.2.1 (The R Foundation for Statistical Computing Platform, https://www.r-project.org)

Conversely, since ferritin and TSAT increased with ALT on day 3, we investigated the relationship between liver dysfunction and iron metabolism. The patient’s ALT and TSAT decreased during hospitalization (Fig. 2A), but a non-parametric test between ALT and TSAT did not indicate a significant correlation (*r*_*s*_ = 0.9, *P* = 8.33 × 10^−2^) (Fig. 2D).

Next, we examined the relationship between Alb and ALT. As mentioned above, we divided ALT and Alb by HCT to eliminate the change by dehydration. ALT/HCT correlated inversely with Alb/HCT in the patient (Fig. 2B), and the non-parametric test indicated a significant correlation (*r*_*s*_ = -0.835, *P* = 5.24 × 10^−5^) (Fig. 2E). By contrast, the iron-storage protein ferritin and the liver enzyme GGT showed an upward trend up to day 15 when adjusted for HCT, which then decreased (Fig. 2C). A non-parametric test between ferritin/HCT and GGT/HCT also suggested a significant correlation (*r*_*s*_ = 1.0, *P* = 2.78 × 10^−3^) (Fig. 2F).

A few days after dietary calorie intake exceeded 1,400 kcal per day, we observed a decrease in the patient’s blood phosphorus levels due to refeeding syndrome (blood phosphorus = 1.4 mg/dL). Accordingly, we performed phosphorus supplementation as needed (inorganic phosphorus 30 mmol per day was administered by intravenous drip from the 9th day, followed by oral preparation 32.3 mmol per day from the 18th day, and gradually decreased and discontinued from the 39th day), in an attempt to maintain the blood phosphorus concentration within normal limits. As for other electrolytes, which tend to decrease during refeeding, we prophylactically administered intravenous fluids containing potassium and magnesium (10 milliequivalents [mEq] and 2.5 mEq per day, respectively). We did not find severe hypokalaemia or hypomagnesemia in her test results. At follow-up four months after discharge, she weighed 56.2 kg (BMI 22.2 kg/m^2^) and noted resumption of menstruation for the first time in 15 months.

## Discussion and conclusions

We presented a patient with AN with elevated TSAT and transaminases, which improved with increased BW, suggesting that an undernutrition-associated decrease in the iron transport protein transferrin might cause IOL, resulting in liver damage. ALT normalized with weight gain, and the correlation between ALT and weight was statistically significant (Fig. 1A, C). TSAT and ALT appeared to correlate well over time, but their correlation was not significant, probably because TSAT was measured only five times (Fig. 2A, D).

Meanwhile, Alb is a well-known surrogate marker of nutritional status, but a recent systematic review casts doubt on its usefulness [6]. Indeed, the correlation with BW appears low, showing the highest value at the lowest BW and the minimum after our patient’s weight started to increase (Fig. 1A, bottom). However, considering the effect of dehydration by dividing the blood test values by HCT, which reportedly correlates well with circulating plasma volume [7], HCT-divided Alb tended to increase in parallel with increasing BW, and we confirmed a strong correlation with non-parametric tests (Fig. 1B, D). For this reason, we decided to divide each blood test value by HCT for further analysis to mitigate the effect of dehydration. Besides HCT, there are various dehydration markers on blood examination. Blood urea nitrogen (BUN) and creatinine are commonly used, but they are affected by other causes unrelated to dehydration, such as protein consumption and muscle mass. Therefore, we think it is not suitable for patients with AN. Conversely, our patient had hypogonadotropic hypogonadism because of malnutrition, which causes cessation of menstruation, making HCT relatively stable. As a result, we selected HCT as a dehydration marker in this case.

In Fig. 1A, the change in weight and ALB do not correlate well at the early stage of hospitalization. Specifically, during the initial ten days of hospitalization, the body weight gradually decreased while the ALB levels increased rapidly until the fourth day and then decreased suddenly. One of the reasons why blood Alb and weight change did not correlate well may be associated with third spacing. Nutritional supplementation was performed with peripheral infusion (210 kcal/500 mL/day) from the 4th day until the 10th day of hospitalization so that the blood Alb might have been diluted due to fluid overload. However, after discontinuing the infusion, the fluid shifted into interstitial space, resulting in a poor correlation between serum Alb throughout the hospitalization period. The change in the slope of Alb in Fig. 1A appears to overlap at the start and discontinuation of the fluid.

Looking at the relationship between nutritional status and liver dysfunction, Alb and ALT were inversely correlated when divided by HCT (Fig. 2B and E), suggesting that improved nutrition may have relieved the patient’s liver dysfunction. The changes in ferritin and GGT adjusted for HCT correlated well (Fig. 2C, F), supporting our hypothesis that a decline in transferrin synthesis may cause liver damage.

The relationship between impaired iron transport and liver damage was well documented by Kim and Leitch [5]. Iron is generally transported in a stable state bound to transferrin. However, with excess iron relative to transferrin, labile NTBI can accumulate in hepatocytes, promoting ROS production and causing liver damage. In patients with AN, a decrease in transferrin owing to undernutrition might lead to IOL, resulting in liver dysfunction. Furthermore, functional hypothalamic amenorrhea, which had been present for nine months, may have contributed to IOL in this patient.

We have deduced the mechanism for why ferritin and GGT increased shortly after her weight gain and then decreased, unlike ALT (Fig. 2D). Ferritin and GGT are involved in detoxification; in particular, ferritin has a critical role in iron detoxification [8–10]. However, malnutrition suppressed ferritin and GGT synthesis, but improving the patient’s nutritional status promoted their synthesis and transferrin production. The elevated transferrin level decreased TSAT and NTBI, which was followed by a reduction in ferritin and GGT.

This study has limitations. First, the report is based on only one patient. Therefore, it is unclear whether this pathology applies to other patients with AN. Second, we believe there was no significant correlation between TSAT and ALT because the number of measurements for TSAT was too small, but this is uncertain. Third, it is unsure whether the elevated TSAT in this patient is associated with labile NTBI and ROS production in her hepatocytes because of the lack of experiments using the patient’s liver biopsy specimen. Fourth, HCT-divided Alb could be more valuable than its raw data as an indicator of changes in the nutritional status of a single patient. However, comparing nutritional status with other patients, especially those who experience bleeding, could be useless because the steady-state HCT levels would differ for each patient. Furthermore, for unknown reasons, Alb/HCT had small fluctuations that did not completely correlate with weight changes. Therefore, when using Alb/HCT as a nutritional marker, we must be cautious about this point.

## Conclusions

We present a patient with AN, with elevated transaminases, GGT, TSAT, and ferritin, which normalized with nutritional improvement. When adjusted for dehydration by HCT, Alb and BW correlated significantly, and there were significant correlations between ALT and Alb and between ferritin and GGT. Defective iron utilization might be the cause of liver dysfunction in AN. HCT-adjusted Alb could be a better marker for estimating nutritional status.

## Data Availability

The datasets generated and/or analysed during the current study are available from the corresponding author on reasonable request.

## Abbreviations

Alb: Albumin
ALT: Alanine aminotransferase
AN: Anorexia nervosa
BMI: Body mass index
BUN: Blood urea nitrogen
Cre: Creatinine
GGT: Gamma-glutamyl transferase
HCT: Haematocrit
IOL: Iron overload
NTBI: Non-transferrin-bound iron
ROS: Reactive oxygen species
TSAT: Transferrin saturation
